# Auguries of adaptivity: LES γδ TCR ligand recognition revisited

**DOI:** 10.1016/j.it.2025.10.006

**Published:** 2025-12-01

**Authors:** Juliet L. Gunn, Anzelika Rubina, Ceri A. Fielding, Fiyaz Mohammed, Eddie C.Y. Wang, Carrie R. Willcox, Benjamin E. Willcox

**Affiliations:** 1 Department of Immunology and Immunotherapy, School of Infection, Inflammation and Immunology, College of Medicine and Health, University of Birmingham, Birmingham, UK; 2 Cancer Immunology and Immunotherapy Centre, College of Medicine and Health, University of Birmingham, Birmingham, UK; 3 National Institute for Health and Care Research (NIHR), Birmingham Biomedical Research Centre, Birmingham, UK; 4 Division of Infection and Immunity, School of Medicine, Cardiff University, Cardiff CF14 4XN, UK

## Abstract

Identification of antigenic ligands for the γδ T cell receptor (TCR) has remained a highly challenging goal since the emergence in the 1980s of γδ T cells as a distinct immune compartment. In a significant advance more than 12 years ago, endothelial protein C receptor (EPCR), a cell-surface-expressed major histocompatibility complex (MHC)-like protein that binds phospholipids, was identified as the first ligand for a human γδ TCR to be validated by direct binding experiments: a finding that undoubtedly posed more questions than it answered. In this review we discuss how features of this single clonotypic specificity anticipated insights into adaptive-like human γδ T cell biology that emerged in subsequent investigations, and we highlight recent findings about EPCR that point towards the relevance of such responses in anti-pathogen and potentially anti-tumour immunity.

## Human γδ T cells: an exclusively innate-like compartment?

**Figure 1:**
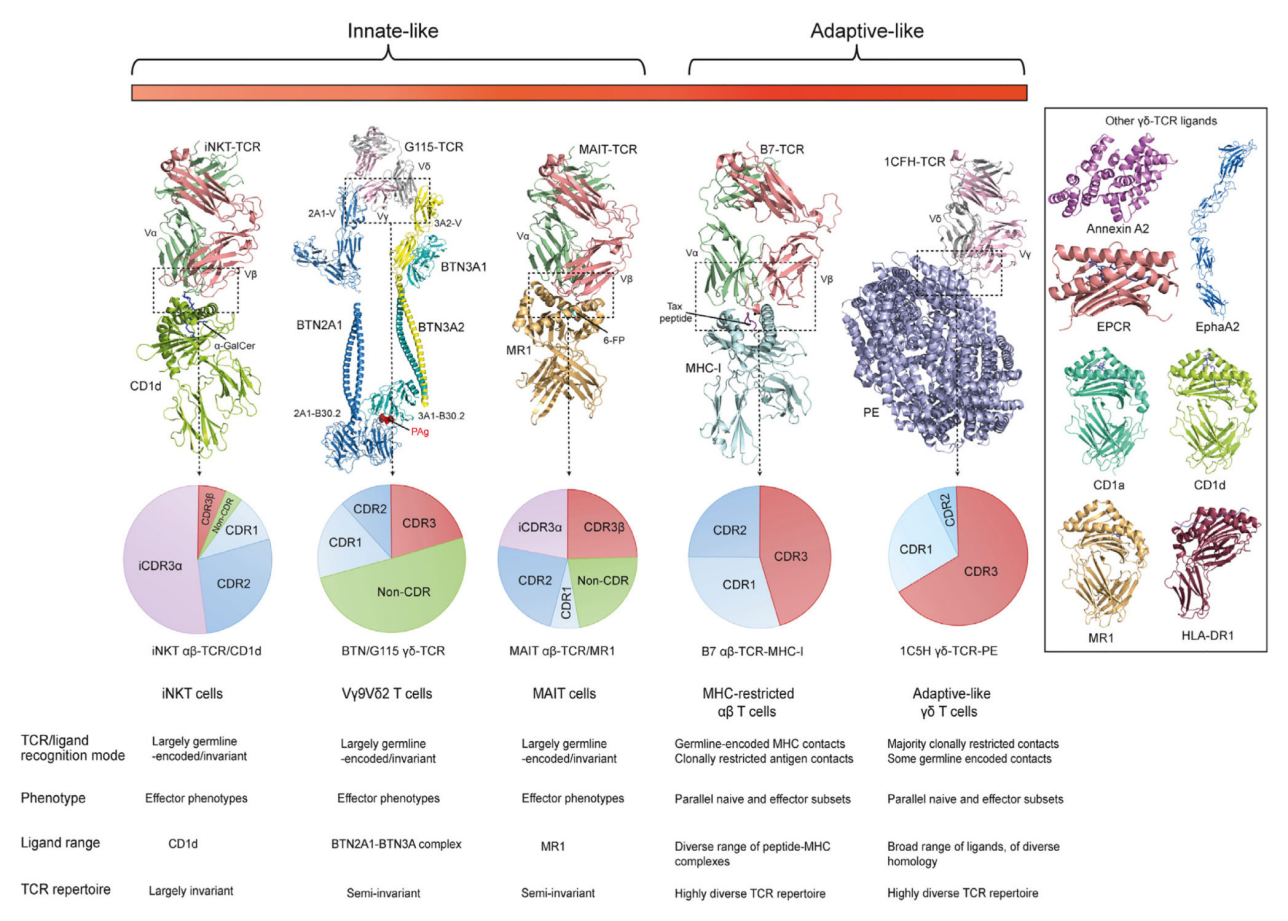
Adaptive-like versus innate-like γδ and αβ T cell recognition. Key differences between innate-like lymphocytes such as invariant natural killer T (iNKT) (αβ) cells, Vγ9Vδ2 T cells, and mucosal-associated invariant T (MAIT) cells (αβ), and more adaptive populations such as conventional major histocompatibility complex (MHC)-restricted αβ T cells, and adaptive-like γδ T cells. Innate-like subsets typically adopt effector phenotypes and utilise more germline-encoded/invariant elements of the T cell receptor (TCR) to engage a restricted set of ligands, whereas adaptive populations retain naïve populations from which clonotypically dependent effector responses targeting a diverse array of ligands can be generated. TCR–ligand complex structures (left to right) relate to PDB codes 2PO6 (iNKT TCR-**CD1d**-α-GalCer); 9JQR (G115 TCR–butyrophilin (BTN)2A/BTN3A), 4L4T (MAIT TCR–MR1); 1BD2 (αβ TCR–MHC class I); and 9O62 (1C5H TCR–phycoerythrin, PE). PDB codes for individual adaptive-like γδ TCR ligands (right-hand side) are 2HYW (Annexin A2); 7OKT (endothelial protein C receptor, EPCR); 2×10 (ephrin type-A receptor 2, EphA2); 1ONQ (CD1a-sulfatide); 1ZT4 (CD1d-α-GalCer); 4GUP (MR1); and 2FSE (HLA-DR1). Pie charts outline the proportion of TCR–ligand contacts (as assessed by contribution to buried surface area of the complex) involving variable CDR3 elements (red), as opposed to CDR1 (light blue), CDR2 (blue), germline-encoded non-CDR elements (green), or invariant CDR3α (iCDR3α; light purple) elements. Variable CDR3 elements are notably decreased for the innate-like (iNKT, Vγ9Vδ2, and MAIT) populations relative to adaptive αβ TCR–peptide–MHC interactions and adaptive-like γδ TCR–ligand interactions (here illustrated by TCR interaction with PE).

**Figure 2:**
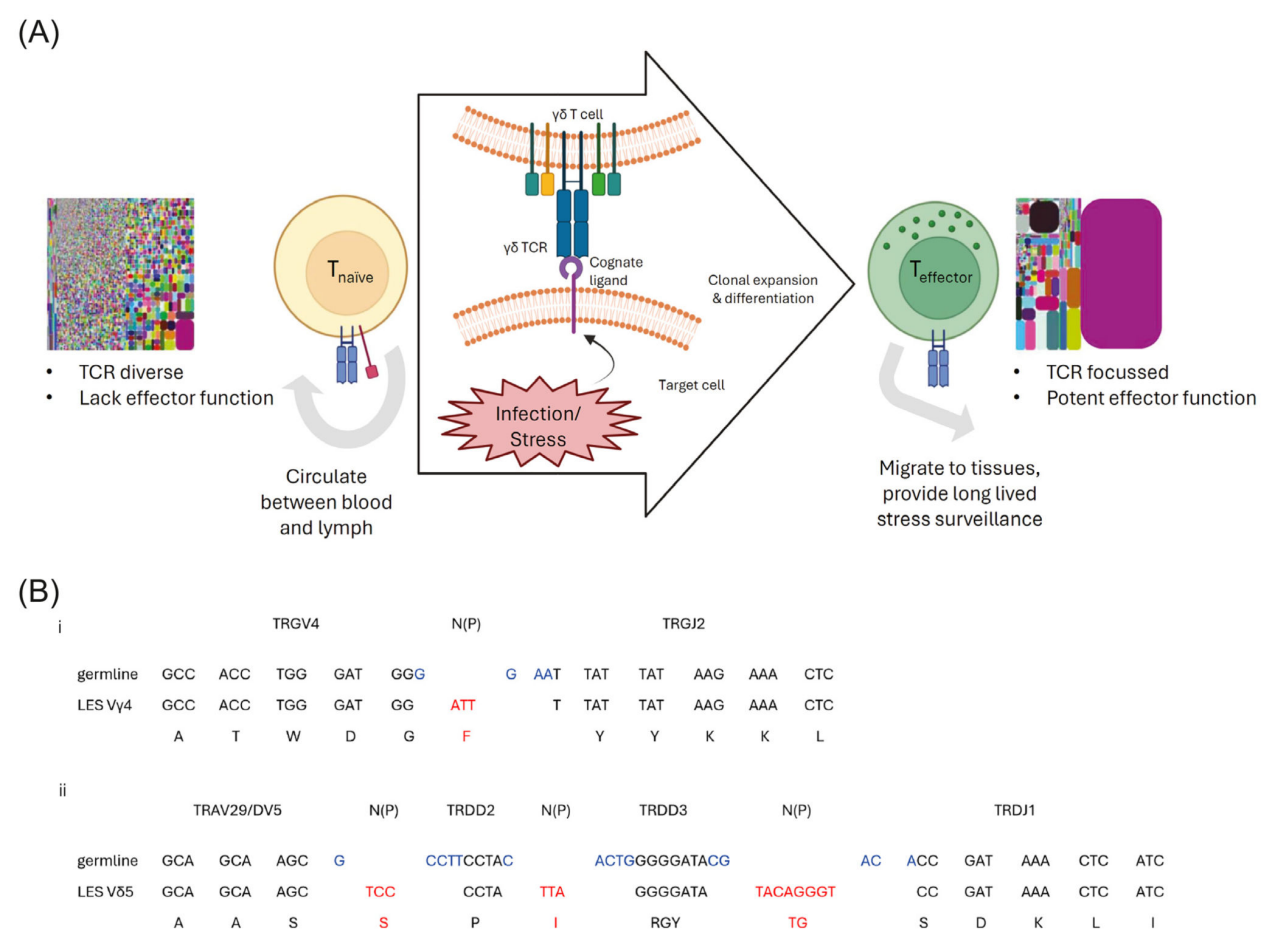
Adaptive-like γδ T cell immunobiology. (A) Vγ9Vδ2-negative γδ T cells are proposed to operate in an adaptive-like mode, whereby in response to infectious or non-infectious stress challenges, particular γδ T cell receptor (TCR) clonotypes capable of recognising physiologically relevant ligands become selectively expanded, with resultant TCR signalling helping to drive a transition from T_naive_ to an antigen-experienced T_effector_ status. This paradigm predicts that expanded γδ TCR clonotypes recognise cognate ligands upregulated or altered during such scenarios, and that this occurs via their CDR3 regions. (B) Recombination of the LES γδ TCR chains, illustrating the private nature of the LES γδ TCR sequence. (i) The LES TCR-γ chain was generated by recombination of TCR-γ variable region 4 (TRGV4) with TCR-γ Joining 2 (TRGJ2). Four nucleotides were removed by exonuclease activity (blue; X = 4) from the ends of the germline gene segments during V(D)J recombination, while three N nucleotides (red; *n* = 3) were added by terminal deoxynucleotidyl transferase (TdT). (ii) The LES TCR-δ chain is complex and private, using two TCR-δ chain diversity (TRDD) segments, with 15 nucleotides removed by exonuclease during recombination (blue, X = 15), and 14 N nucleotides added across the three recombination sites (red, *n* = 14). Figure created with BioRender (https://BioRender.com/inwlfan).

γδ T cells have traditionally been regarded as innate-like lymphocytes. Arguably the best candidate for such a biology in humans is the predominant peripheral-blood γδ lymphocyte subset, Vγ9Vδ2 T cells [1]. This γδ T cell subset is present from early in life [2], with Vγ9Vδ2 T cells emerging from the thymus as pre-programmed effector cells [3], capable of responding *en masse* to bacterially derived pathogen-associated molecular patterns (PAMPs) (see Glossary) such as (E)-4-hydroxy-3-methyl-but-2-enyl pyrophosphate (HMBPP) via interactions of different regions of the semi-invariant Vγ9Vδ2 T cell receptor (TCR) with target cell expressed B7-like **butyrophilin family members** [1]. However, one of the most surprising and intriguing developments in human γδ T cell biology has been the emergence, particularly over the past decade, of a previously unrecognised adaptive-like immunobiology [4–8], which appears to apply to Vγ9Vδ2-negative γδ T cells. While this biology has been reviewed comprehensively elsewhere [4,5,8], a brief summary of key observations is warranted. The paradigm that has emerged has been built substantially on both TCR repertoire [5,6,9,10] and phenotypic data [5,6,9] that highlight both TCR diversity, phenotypically distinct T_naive_ and T_effector_ subsets, and the potential for highly focused clonotypic expansion and differentiation in response to infection [10–12]. These features differ from those of unconventional innate-like T cell populations such as invariant natural killer T (iNKT) cells, mucosal-associated invariant T (MAIT) cells, and Vγ9Vδ2 T cells, and in some respects more closely align with conventional major histocompatibility complex (MHC)-restricted αβ T cell populations, albeit differing radically with respect to ligand recognition (Figure 1). Importantly, such Vγ9Vδ2-negative γδ T cells are also proposed to operate in an adaptive-like mode (Figure 2A), whereby in response to infectious or non-infectious stress challenges, particular γδ TCR **clonotypes** capable of recognising physiologically relevant ligands become selectively expanded, with resultant TCR signalling helping to drive a transition from T_naive_ to an antigen-experienced T_effector_ status. This paradigm predicts that expanded Vγ9Vδ2-negative TCR clonotypes recognise cognate ligands upregulated or altered during such scenarios, and that this occurs via their CDR3 regions.

Currently, an understanding of how this immunobiology operates for individual γδ TCR ligands is largely lacking. This remains arguably the most critical unanswered question in the field, and one that, if addressed, could unlock understanding of – and also ultimately therapeutic exploitation of – broadly applicable MHC-unrestricted γδ TCRs that enable sensing of stress, infection, and transformation. In this review we attempt to probe this question, with reference to the canonical LES–EPCR receptor–ligand system, the first human γδ-TCR–ligand interaction to be directly validated [13].

### Origin and cellular reactivity of the LES clone

Before the LES-endothelial protein C receptor (EPCR) interaction was characterised, several studies suggested that the human γδ compartment could be delineated into Vγ9Vδ2 T cells that were responsive to phosphoantigens (P-Ags) [14], and a more TCR-diverse Vδ2^neg^ compartment that was not. While Vδ2^neg^ T cell immunobiology was unclear, seminal work indicated relevance to human cytomegalovirus (HCMV) [15]: a pathogen which, although highly immunogenic – resulting in a distinct signature on peripheral blood lymphocytes in healthy seropositive subjects – maintains lifelong persistent infection and can drive morbidity and mortality in immunosuppressed scenarios. Although Vδ2^neg^ subsets displayed certain features characteristic of conventional adaptive immunity, this was against a backdrop of the emergence of specific innate-like lymphocyte populations such as iNKTs [16] and MAITs [17], which exhibited clear effector capacity from early life combined with semi-invariant TCR usage (Figure 1). Moreover, analogous findings in the mouse γδ T cell compartment fuelled speculation that human γδ T cells might be exclusively innate-like in function [16,17].

The LES specificity that recognises EPCR emerged from studies of human HCMV infection, which drives increased Vδ2^neg^ T cell numbers in peripheral blood, including following solid organ transplantation [18,19]. Halary *et al*. derived cytotoxic Vδ2^neg^ T cell clones from HCMV-exposed individuals, the LES clone originating from an individual with acute HCMV infection after lung transplantation [20]. A notable feature of such clones, including LES, was TCR-dependent dual reactivity against HCMV-infected target cells (typically fibroblasts), and various tumour cell lines [20]. Interestingly, different T cell clones exhibited distinct patterns of tumour cell reactivity. One scenario envisaged to explain this phenomenon involved γδ TCR cross-recognition of distinct but homologous HCMV-encoded and host-encoded targets upregulated on HCMV-infected and tumour cells, respectively. A second, arguably simpler, explanation involved γδ TCR-mediated recognition of individual host-encoded stress ligands induced by both HCMV infection and upon tumourigenesis.

### Identification of EPCR as a ligand for the LES γδ TCR

Willcox *et al*. employed an immunisation strategy to generate a blocking antibody (2E9) that bound target cells and selectively abrogated LES T cell recognition [13]. Immunoprecipitation from target cells using 2E9 enabled identification of the LES γδ TCR ligand. This technically challenging approach has been used subsequently to identify ligands for other Vδ2^neg^ γδ TCR specificities [7,21,22], and is less biased than some other approaches such as tetramer staining [7].

Subsequent mass spectroscopy analysis of 2E9 immunoprecipitates revealed the candidate ligand to be EPCR [13]. EPCR – an MHC-like type-1 transmembrane cell-surface protein consisting of an α1–α2 lipid antigen-binding platform linked to a transmembrane region – regulates the clotting cascade by binding to activated protein C [23]. Notably, EPCR is expressed on endothelial cells, a significant target of HCMV infection *in vivo*, consistent with a role for γδ T cells in surveillance of this cellular niche. *In vitro* experiments using 2E9 indicated that γδ TCR engagement of EPCR was functionally critical to LES T cell recognition of both HCMV-infected cells and EPCR^+^ tumour cells. This validated the second scenario outlined earlier, involving γδ TCR-mediated recognition of a host-encoded ligand present in both infection and tumourigenesis. Ultimately, recombinant EPCR was shown to bind LES γδ TCR directly via surface plasmon resonance, with a relatively low affinity (80–100 μM), which represented a crucial confirmation of EPCR as a direct γδ TCR ligand [13].

### LES–EPCR interaction: a molecular exemplar of adaptive-like stress recognition

The aforementioned findings raised many questions, specifically in four areas discussed in the following sections.

#### ‘Multimolecular stress signature’ recognition

Curiously, LES γδ TCR–EPCR engagement was necessary but insufficient for target cell recognition [13]. Notably, certain tumour cell lines expressed substantial cell-surface EPCR levels, but did not support recognition by the LES T cell clone or LES–TCR–JRT3 reporter cells. Initially, EPCR on activating cell lines (and HCMV-infected cells) was hypothesised to present a specific lipid recognised by the LES γδ TCR. However, this would necessitate TCR binding to the lipid-presenting surface of the α1–α2 platform, whereas mutagenesis indicated recognition of its opposite side [13]. Instead, it appeared that LES–γδ TCR–EPCR recognition must be complemented by recognition of TCR-extrinsic factors, with CD2/CD58 and leukocyte-function-associated antigen 1 (LFA-1)–intercellular adhesion molecule 1 (ICAM-1) co-stimulatory receptor–ligand axes emerging as important components of a ‘multimolecular stress signature’ [13]. In the context of HCMV, infection increases ICAM-1 expression but decreases CD58 expression [24,25]. The LES–EPCR interaction established a precedent for a Vδ2^neg^ γδ TCR recognising an MHC-like molecule via a highly unusual binding mode compared with classical αβ TCR–pMHC interactions or even αβ TCR–CD1 interactions, one subsequently extended to **MR1** recognition [26,27]. It also underlined the importance of integration of TCR-dependent and extra-TCR stress signals to γδ T cell activation.

#### The LES–TCR clonotype: entirely private rather than semi-invariant

A second major question was whether the LES–TCR exemplified a semi-invariant EPCR-specific subset. Importantly, the Vγ4Vδ5 LES–TCR chain usage was highly unusual within the Vδ2^neg^ population, unlike semi-invariant human iNKT cells that typically express Vα24–Jα18 paired with Vβ11 TCR chains, or human MAIT cells that express Vα7.2–Jα33 TCRα chains and preferentially pair with Vβ2/Vβ13 TCR chains [16,17]. Therefore, a widespread semi-invariant EPCR-reactive population was not evident, and functional Vδ2^neg^ T cell reactivity to EPCR was not detected in other individuals [13]. This suggested the LES–TCR might be a private reactivity to EPCR; consistent with this, the LES δ-TCR chain incorporated numerous N/P-nucleotides, underlining the LES γδ TCR as a private clonotype [13] (Figure 2B).

#### An emergent adaptive-like immunobiology for the Vδ2^neg^ T cell subset

The findings outlined above prompted the somewhat speculative suggestion that the LES γδ TCR reactivity was ‘unique but paradigmatic’ [13], which has ultimately proved prophetic. Subsequent studies established three apparent cornerstones of adaptive-like γδ T cell immunobiology.

First, such subsets have a private TCR repertoire underpinned by very high diversity in Vδ-CDR3, due to extremely high N/P region addition, substantial exonuclease nibbling, and potential for multiple D-segment incorporation [6]. Based on their Vδ genes alone, most Vδ2^neg^ clonotypes represent ‘one-off’ recombination events. A second cornerstone relates to clonal expansion, which is evident both in peripheral blood [6,10] and tissue-associated adaptive-like γδ T cell populations [9], and is linked to infection, including with HCMV [10,11]. A third relates to phenotypic differentiation of adaptive-like γδ T cells, aligned to clonal amplification [6,11]. Such populations appear to be produced in a T_naive_ state (CD27^hi^ TCF7^+^) that broadly phenocopies CD8^+^ T_naive_ cells, lacks effector markers, and expresses homing receptors (e.g., CCR7, CD62L) compatible with circulation between blood and lymph [4–6,11]. Importantly, such T_naive_ γδ T cell populations are highly TCR diverse. By contrast, expanded Vδ2^neg^ or Vγ9^neg^Vδ2 clonotypes reside entirely in T_effector_-like (CD27^lo/neg^) populations that typically express cytotoxic markers (perforin, granzyme), are cytotoxic and produce cytokines, have upregulated peripheral homing markers (e.g., CX3CR1) [4–6,11], and express transcription factors associated with effector status including chiefly eomesodermin (EOMES) and T-bet [12]. Importantly, pathogen infection, including HCMV infection, drives not only clonal expansion but also phenotypic transition from T_naive_ to T_effector_ status [11,12].

These features suggest that non-Vγ9Vδ2 γδ T cells can operate in a CDR3 and ligand-dependent adaptive-like mode (Figure 2A). Reassessment of LES clonotype features indicates that it aligns closely with this adaptive-like paradigm, and specifically with a physiological T_effector_ clonotype. First, the private nature of the LES clonotype reflected the adaptive-like γδ T cell repertoire as a whole. The high CDR3 N/P region addition within the LES Vδ5-CDR3 is closely matched to that of the entire Vδ2^neg^ repertoire (14 N/P for LES Vδ5, versus an average of ~15 for TCR-Vδ) [6], indicating extreme privacy. For a typical Vδ2^neg^ clonotype, based on N/P nucleotide addition alone, the chances of recombining an identical nucleotide sequence will be <1 in a billion (4^15^). Second, the LES clonotype was heavily clonally expanded following *in vivo* HCMV infection (to 25% of peripheral blood T cells), and third, the LES cellular phenotype (CD28^neg^, CD45RO^neg^) and cytotoxic capabilities clearly delineated it as a T_effector_ cell [28]. Also consistent with this adaptive-like paradigm, recognition of EPCR by the LES γδ TCR has been shown to be highly CDR3-dependent, both for TCR-Vγ and TCR-Vδ [13,29].

#### Re-evaluation of EPCR as a stress-induced ligand

Although the aforementioned considerations suggest that LES–EPCR recognition may reflect a physiological cognate antigen-specific γδ T cell response, an additional question concerned EPCR’s credentials as a stress-induced ligand. At the time of the initial ligand discovery [13], it was largely unclear whether EPCR was upregulated upon infectious or non-infectious stress, and if so, why. Subsequent studies have shed light on this issue, in both tumour and HCMV infection settings. Initially, EPCR was observed to be overexpressed on certain tumour cell lines, but the underlying reason was unclear, and upregulation on primary tumours was not well established. Lal *et al*. showed that EPCR overexpression was related to gene amplification and DNA hypomethylation, which occurred in various epithelial cancers alongside several adjacent genes on chromosome 20q, a region previously implicated in chemoresistance [13,30]. Moreover, EPCR protein overexpression was routinely observed in primary colorectal cancers [13,30]. Thus, EPCR can legitimately be considered a molecular marker of tumour-associated alterations in epithelial cancers.

**Figure 3:**
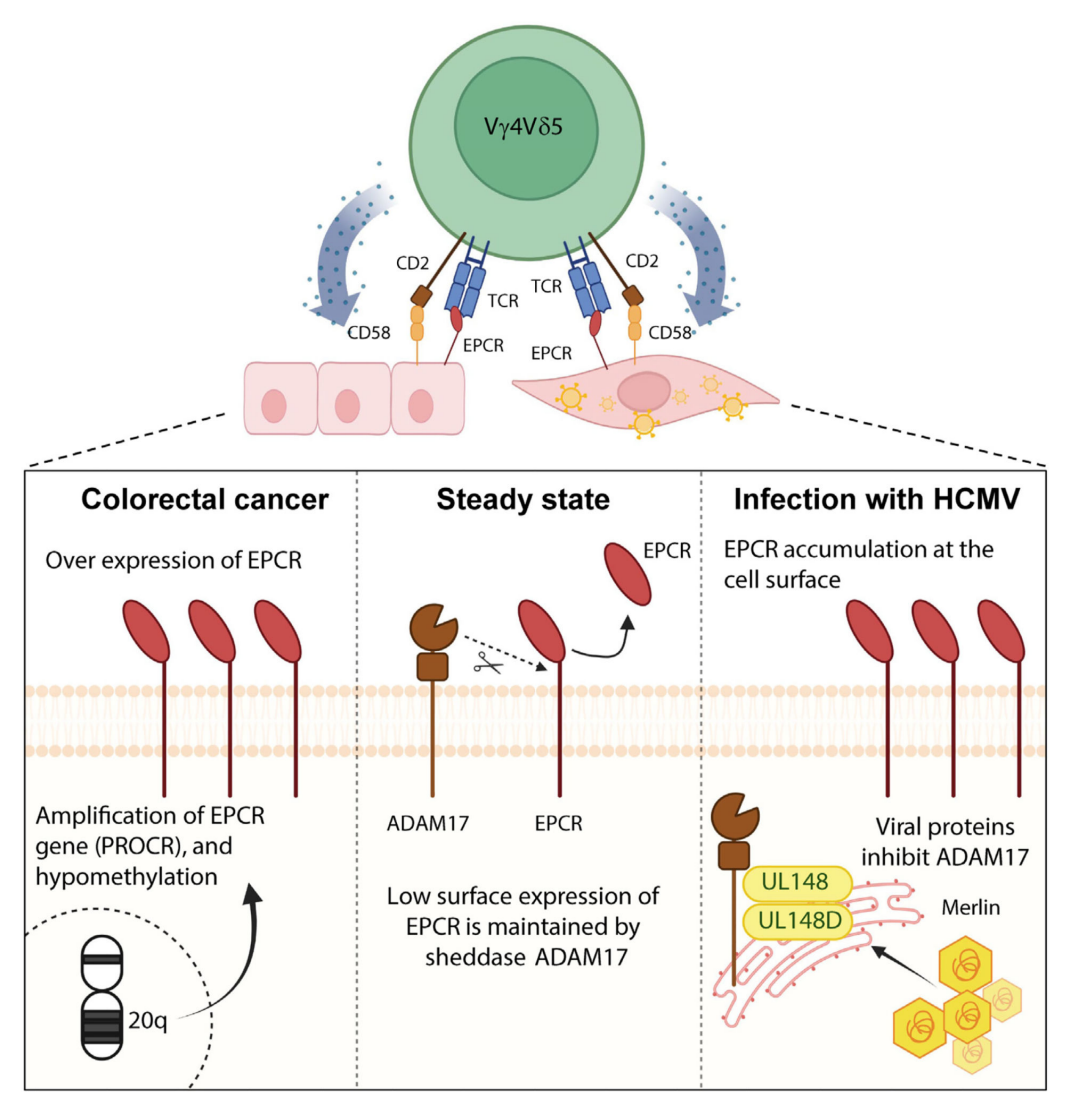
Dysregulation of endothelial protein C receptor (EPCR) in microbial and non-microbial stress. Dysregulation of EPCR expression in human cytomegalovirus (HCMV) infection (right) and cancer (left) compared with the steady state (centre). Centre: cell surface EPCR levels in the steady state are limited by the activity of ADAM-17, which cleaves the EPCR ectodomain. Right: infection of fibroblasts with Merlin HCMV strain leads to downregulation of the cell surface ‘sheddase’ ADAM-17, leading to increased accumulation of EPCR at the cell surface. Left: increased EPCR in cancer cell lines and primary cancer cells is underpinned by chromosomal amplification and demethylation at 20q, and is observed in primary colorectal cancer tissue. Abbreviations: TCR, T cell receptor; ADAM17, a disintegrin and metallopeptidase 17. Figure created with BioRender (https://BioRender.com/j8hmzpc).

Intriguingly, early studies highlighted that although HCMV infection of target cells sensitised them for TCR-dependent and EPCR-dependent recognition by LES γδ T cells, EPCR expression level itself was unaltered by HCMV infection [13]. This cast doubt on whether EPCR is a genuine infectious stress ligand, and suggested instead that EPCR might alternatively represent a restriction factor facilitating γδ T cell immunosurveillance of the endothelial niche, with HCMV-induced TCR-extrinsic factors underlying induction of LES activation [13]. However, an important caveat is that these studies utilised a strain of HCMV (TB40/E) for which the clinically derived virus exists as a mix of variants, containing some sequence variation in both genes that are considered ‘hypervariable’ and those less variable, whilst maintaining the full range of HCMV cellular tropism [31]. However, when the Merlin HCMV strain was used to infect target cells, cell surface EPCR expression was substantially upregulated [32]. Analysis of a complete library of Merlin strain single-gene deletion mutants covering the entire U_L_/b′ region demonstrated that EPCR upregulation was dependent on the viral UL148 and UL148D genes (Figure 3) [32]. Proteomic plasma membrane profiling revealed that UL148/UL148D genes stabilised surface expression, not only of EPCR, but of >100 proteins, the vast majority of which were host-encoded. This was dependent on UL148/UL148D-mediated inhibition of the maturation of a disintegrin and metallopeptidase 17 (ADAM17), the prototypic ‘sheddase’, which would otherwise cleave numerous membrane-associated proteins, including EPCR [33], to release their ectodomains extracellularly. Viral targeting of ADAM-17 also modulated expression of proinflammatory cytokine receptors such as tumour necrosis factor receptor 1 (TNFR1) and TNFR2 but, perhaps more significantly, also resulted in evasion of NK cells during infection, probably through stabilisation of as yet unidentified inhibitory NK ligands [32]. This study therefore establishes that, at least in the context of certain viral strains and target cell niches, not only can EPCR expression become up-regulated upon infectious stress during HCMV infection, but this upregulation may represent a molecular flag indicative of an important viral immunoregulatory mechanism in target cells.

**Figure 4:**
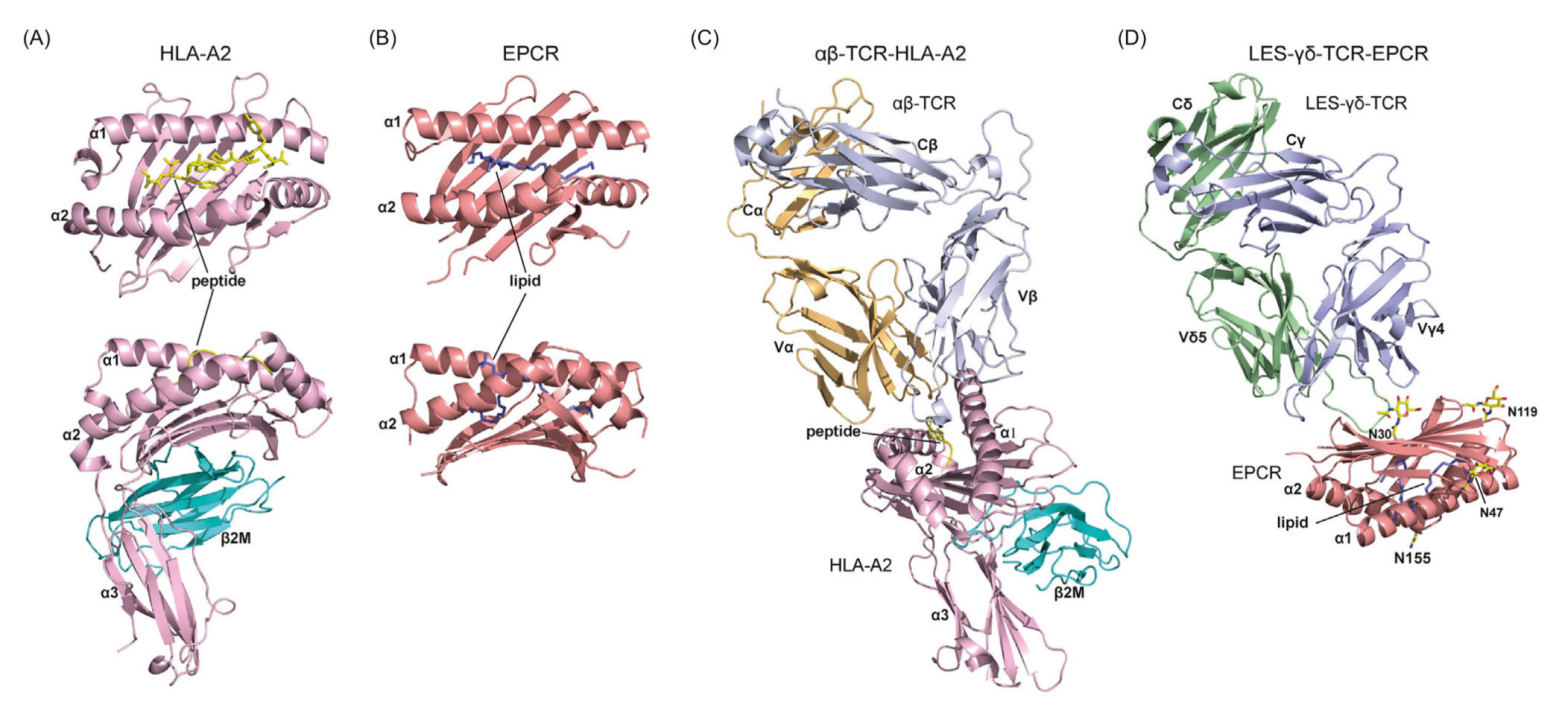
Divergent recognition mechanisms of αβ and γδ T cell receptors (TCRs) for major histocompatibility complex (MHC) and endothelial protein C receptor (EPCR). (A) Crystal structure of the human leucocyte antigen (HLA)-A2 molecule in complex with the human T-lymphotropic virus type 1 (HTLV-1) Tax peptide (PDB ID: 1BD2 [48]). A ribbon diagram shows HLA-A2 composed of α1 and α2 domains forming the antigen-binding platform, with the α3 domain non-covalently associated with β_2_-microglobulin (β2M) (bottom panel). The HTLV-1 Tax peptide is nestled within the peptide-binding groove between the α-helices (top panel). (B) Crystal structure of EPCR bound to lipid (PDB ID: 7OKT [49]). A ribbon diagram highlights the MHC class I-like architecture of EPCR, formed by α1 and α2 domains (bottom panel). EPCR lacks an α3 domain and does not associate with β2M. The lipid sits deeper within the EPCR antigen-binding groove compared with peptides bound to HLA-A2. (C) Crystal structure of a human αβ TCR (B7 TCR) in complex with HLA-A2 presenting the HTLV-1 Tax peptide (PDB ID: 1BD2). The αβ TCR engages the HLA-A2 antigen-binding platform in a diagonal docking orientation via its complementarity-determining region loops. (D) High ambiguity driven protein–protein docking (HADDOCK)-derived mutagenesis-informed model of the LES γδ TCR bound to EPCR. The LES γδ TCR is predicted to interact in a CDR3-dependent fashion with the ‘underside’ β-sheet of the EPCR antigen-binding platform, in stark contrast to conventional αβ TCR interactions with the α1–α2 domain helices and the peptide moiety of peptide–MHC molecules. N-linked glycosylation sites on EPCR are highlighted.

Although these points relate to quantitative upregulation of EPCR, it is still possible that qualitative changes in EPCR underly antigenicity for LES–TCR interaction in both HCMV and tumour settings. Conceivably, EPCR-intrinsic qualitative changes could explain why its expression is necessary but insufficient for LES–TCR-mediated activation. While the LES γδ TCR is thought to recognise the ‘underside’ of the EPCR platform – disfavouring recognition of putative activatory lipid species, as previously investigated – this mode could conceivably enable the sensing of changes in glycosylation (Figure 4), which have been noted to take place in both HCMV infection [34] and tumourigenesis [35]. This critical area is a priority for future investigations. Specifically, defining the impact of qualitative changes in EPCR on LES–TCR recognition may help to explain the molecular basis of LES–γδ TCR-mediated dual reactivity to infectious stress and transformed self, might have broader significance across adaptive-like γδ T cells, and could have therapeutic implications.

## Concluding remarks

**Figure 5:**
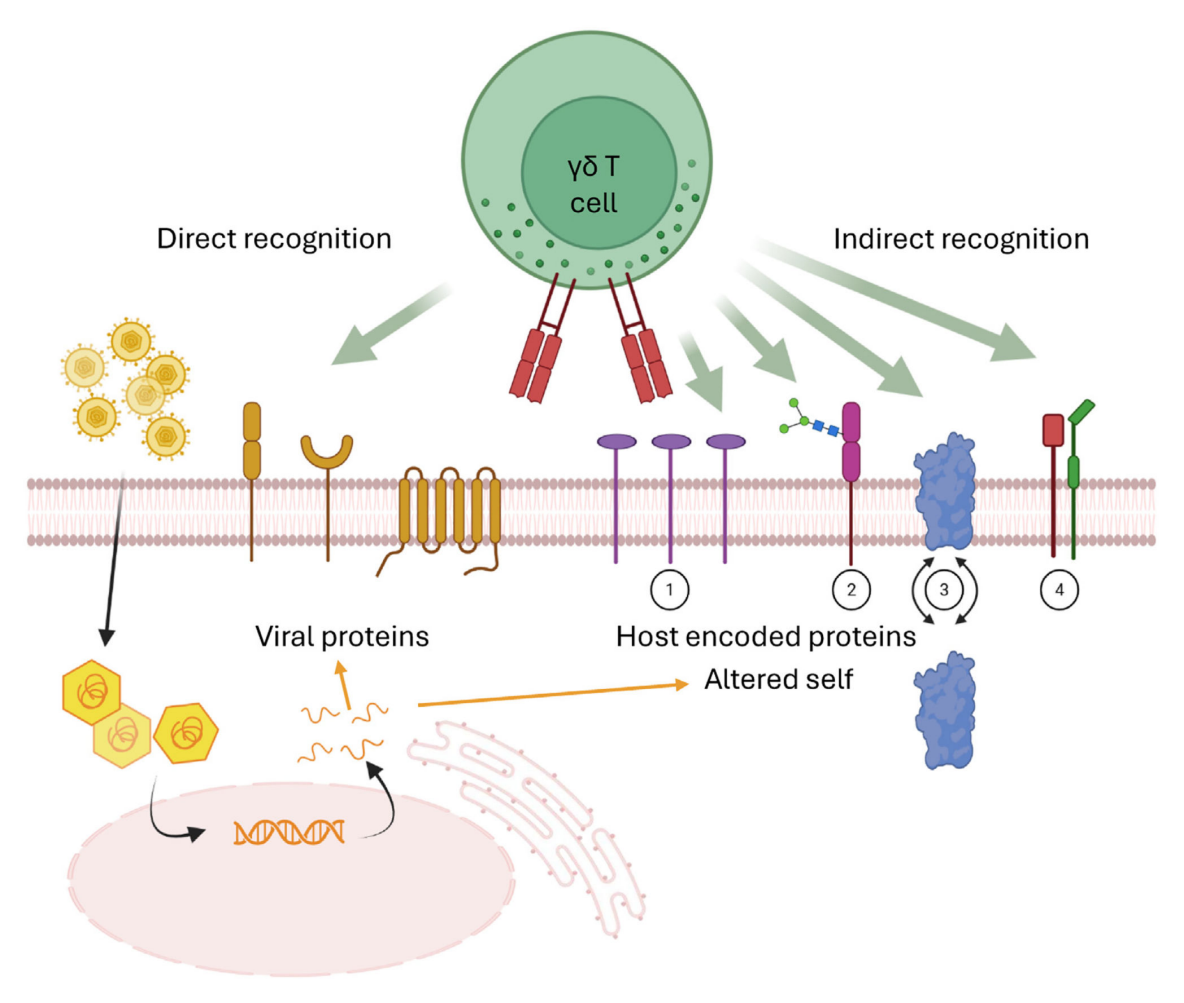
Direct versus indirect γδ T cell recognition of viral infection. γδ T cells could in principle sense viral infection of target cells via direct γδ T cell receptor (TCR) recognition of viral proteins at the cell surface (left arrow); however, some viruses limit the number, expression level, and immunogenicity of viral proteins present at the cell surface. Alternatively, clonal selection from the adaptive γδ TCR repertoire may enable γδ T cells to sense ‘virally altered self’ via changes to host proteins at the cell surface. This could include upregulated levels ①, altered post-translational modification ②, aberrant localisation ③ or dysregulated association status ④ of host-encoded proteins. Figure created with BioRender (https://BioRender.com/67hckti).

A revised interpretation of LES–EPCR interaction as an exemplar of the emergent adaptive-like γδ T cell immunobiology has several implications, and provides a perspective from which to start to address some of the major unresolved questions in the field (see Outstanding questions). First, it seems likely that HCMV will induce γδ TCR-mediated reactivities to other infectious stress ligands. The relatively large number of host-encoded cell surface proteins upregulated following HCMV infection represent a pool of potential targets for such responses [24]. γδ TCR reactivities likely combine with TCR-extrinsic receptor–ligand axes to enable γδ T cell sensing of multimolecular stress signatures indicative of HCMV infection. The challenge of eliciting a viable reactivity from such an extremely diverse TCR repertoire, without the benefits of somatic hypermutation as occurs in antibody generation, is likely high. While HCMV-encoded proteins themselves may in principle be viable targets for such responses, HCMV limits the number of virally encoded cell surface targets, which also tend to be expressed at low levels [24]. Consequently, for HCMV infection, adaptive γδ T cell responses to ‘pathogen-dysregulated self-may be more likely than direct recognition of viral proteins (Figure 5). How thymic development of Vδ2^neg^ T cells might affect affinity/avidity thresholds required for such peripheral γδ T cell activation events is currently unclear, as is the importance of peripheral tolerance mechanisms. These remain some of the most significant unresolved questions in the field, and additional studies on human γδ T cell thymic development and peripheral regulation are warranted. Nevertheless, a prediction of the adaptive-like paradigm is that adaptive-like γδ T_effector_ populations clonally expanded *in vivo* following HCMV infection will be the source of cognate antigen-specific γδ TCR reactivities in this setting. Building on the canonical example of LES–EPCR, harnessing a range of such clonotypes to identify their corresponding physiologically relevant virus-linked stress ligands will be both non-trivial and a major achievement, either for HCMV or for any other pathogen, and could feasibly shed light on common axes of γδ T cell immunosurveillance, as well on the diversity of associated cognate ligands.

A second consideration is what added value adaptive-like γδ T cell recognition provides beyond αβ T cells and NK cells. Its MHC-independence, and focus on TCR ligation of intact cell surface stress antigens, suggest that adaptive-like γδ populations may be analogous to ‘Nature’s chimeric antigen receptor T (CAR-T) cells’ [8]. In the context of HCMV, adept at both suppression of peptide–MHC antigen presentation and NK effector responses [36,37] (Box 1), these features may provide a highly advantageous third arm of cellular immunity less susceptible to immune evasion. A major unresolved question is whether γδ T cell responses are focused on pathogen-derived proteins or altered self-components. Given the limited number and extent of HCMV-encoded proteins expressed at the cell surface, a focus of adaptive-like γδ T cells on recognition of the plethora of HCMV-altered self-antigens, seems paradigmatically more likely, and is exemplified by LES–EPCR [24]. Moreover, the immunological relevance likely extends beyond HCMV. Recent work highlights pronounced adaptive Vδ1 T cell responses in malaria, and this could imply adaptive-like γδ T cell immunosurveillance of the MHC-deficient erythroid niche targeted by this parasitic infection [38]. Also, various other infections have been associated with expansions in adaptive-like γδ T cell populations [8] and could be highly relevant.

An important unresolved question is how subsequent interactions identified between antigenic ligands and adaptive-like γδ TCRs map onto LES γδ TCR–EPCR recognition. These include γδ TCR interactions with diverse self-proteins – CD1 molecules [8], Annexin A2 [8], ephrin type-A receptor 2 (EphA2) [21], MR1 [39] – and even of foreign proteins such as phycoerythrin (PE) [40–42]. In alignment with LES γδ TCR–EPCR interaction, recognition of such ligands appears to be highly clonotypically restricted [7,8]. However, a note of caution: many systems lack information regarding clonotype frequency (e.g., the extent of *in vivo* clonotypic expansion or lack thereof), and regarding cellular phenotype (e.g., T_naive_ versus T_effector_), even if TCR ligand binding is established [7]. In many cases it is also unclear whether clonotypes were expanded *in vivo* following physiological immune challenge (e.g., infection), or expanded during *in vitro* culture [7]. Availability of such information for the LES γδ TCR–EPCR system means that it is uniquely positioned to shed light on how adaptive-like biology operates at a molecular level. An additional point, previously highlighted, is that some antigens (CD1, MR1, PE) were ‘pre-selected’ using recombinant multimer staining reagents, a highly biased method of ligand identification [7]. One intriguing specificity, derived from a *bona fide* CMV-associated clonotypic expansion following allogeneic stem cell transplantation, was found to display some recognition of HLA-DR, and might in principle reflect sensing of elevated class II MHC levels on target cells during CMV infection [43]. However, caveats remain about alignment of this specificity to γδ TCR-mediated recognition of CMV-induced altered self, not least because of the requirement for a non-physiological CDR3 mutation for full HLA-DR recognition, and indeed for any TCR-mediated CMV reactivity, and uncertainty about the role of haplotypic differences, peptide presentation, or other modifications in HLA-DR for recognition by the physiological clonotype [43]. In summary, further studies are imperative to broaden our molecular understanding of adaptive-like γδ TCR sensing of altered self, ideally exploiting systems that mitigate critical experimental limitations.

A final important issue relates to the potential for adaptive γδ T cell responses not just to infection but to cancer. There is considerable interest in unconventional T cell recognition of tumours, particularly since cancer-specific MHC-unrestricted TCRs (αβ or γδ) may in principle be therapeutically applicable to a broad range of patients [44,45]. Although the extent to which dual reactivity to both infection and cancer applies to adaptive-like γδ T cells is unclear, this phenomenon, exemplified by LES–EPCR, will be favoured by a focus on altered self (rather than pathogen-encoded) components that may also become dysregulated in diverse scenarios [13,20]. Cancer-specific γδ TCRs could usefully broaden cell therapy or biotherapeutic approaches given the challenges of identifying safe and selective CAR-T target antigens, the inherent limitations imposed by MHC restriction in the conventional αβ T cell space, and the patient-specific nature of many MHC-restricted neoantigens. Once suitable γδ TCR specificities are in place, both γδ TCR gene transfer-based cellular therapies and development of γδ TCR-based bispecifics would be modalities of interest [45]. However, critical challenges include identification of relevant disease settings, specific patient groups, and ultimately individual γδ TCRs and cognate cancer-specific targets. Understanding the exact nature of such cognate ligands, either in cancer or in infectious settings, how they are dysregulated either qualitatively or quantitatively to elicit γδ TCR recognition, the cellular niches they apply to, and the dynamics of the responses they generate are important future aims in the field. Another crucial issue for tumour targeting is whether relevant cancer targets are fixed in all tumour cells, or, driven by tumour heterogeneity, selectively present in tumour subregions. Recent studies on colorectal cancer [46] and melanoma [47] indicate the potential importance of Vδ2^neg^ γδ T cells in clinically important anti-tumour responses to immune checkpoint blockade, including in MHC-deficient and low mutational tumour burden settings poorly served by conventional αβ T cell responses [47]. Understanding whether such responses are underpinned by TCR-mediated adaptive-like recognition or a parallel NK receptor-mediated reactivity by this same Vδ2^neg^ subset is currently unclear and a focus of ongoing studies.

## Box 1 Evasion of adaptive and innate cellular immunity by CMV

HCMV is well established as a paradigm for viral evasion of αβ T cell and NK cell immunity. A member of the herpesvirus family, its double-stranded DNA genome of 236 kilobase pairs encodes approximately 170 canonical protein-coding genes. The genome is divided into unique long (UL) and unique short (US) regions flanked by repeats, which alongside the terminal repeat left (RL) region, have led to the gene designations.

Since the late 1990s HCMV-encoded proteins have been known to inhibit cell-surface expression of class I MHC molecules (MHC-I). Multiple gene products either drive MHC-I degradation by the proteasome (US2, US11), block its transport to the cell surface (US3), or interfere with peptide loading by inhibiting the transporter associated with antigen processing (TAP) (US6) [50–54]. Subsequent studies have identified other T cell-directed inhibitory effects, including: on peptide processing (mIR-US4-1) [55], a viral herpesvirus entry mediator (HVEM) homologue that triggers the inhibitory B and T cell lymphocyte attenuator (BTLA) (UL144) [56,57], a viral interleukin-10 homologue (UL111A) [58,59], direct triggering of CD45 (UL11) [60], inhibition of T cell activation by upregulation of CEACAM1 (HCMV gene not known [24]) and also by an unknown mechanism for an identified HCMV gene (UL10) [61], and interference with immunological synapse formation through actin manipulation (UL135) [62] or reduction of CD58 expression (UL148) [25].

Downregulation of surface MHC-I should confer sensitivity of HCMV-infected cells to natural killer (NK) cell-mediated cytotoxicity, in line with the ‘missing self’ hypothesis of NK activation. However, HCMV encodes 24 identified NK immune evasins to date (reviewed in [37]). These act in multiple ways, either directly triggering NK cell inhibitory receptors, preventing cell-surface expression of activating ligands for NK cells by retaining them intracellularly, or targeting them for proteasomal/lysosomal degradation, interfering with co-stimulatory or adhesion molecules or via effects on actin polymerisation within infected cells [37]. CMV also downregulates other immune-related molecules such as MR1 [63], CD1 [64], and BTN2A1 [65], although the functional consequences of this are unclear.

HCMV likely encodes further undefined immune evasion mechanisms, as there are still many HCMV ‘orphan’ genes with no defined function or mechanism of action. Furthermore, many HCMV genes are multifunctional, thus additional functions for genes with ‘known’ function may yet be revealed. Despite the plethora of immune evasion genes, and although infection is lifelong, in an immunocompetent host the virus is controlled such that overt disease rarely occurs. Evolution of HCMV with its human host has been ongoing since speciation and it seems that, at least in individuals with intact immunity, the virus and host have reached a ‘détente’. Significant HCMV disease results only when this balance is disrupted: either when HCMV infects individuals with a genetic, viral, or treatment-induced immunosuppression, or in an immature (e.g., foetal) immune system.

## Highlights

The LES γδ T cell clone emerged during human cytomegalovirus (HCMV) infection and responds to HCMV-infected target cells and some cancer cells via recognition of endothelial protein C receptor (EPCR), a major histocompatibility complex (MHC)-like protein that binds lipids.

LES γδ T cell receptor (TCR) clonotype features foreshadowed several features of adaptive-like γδ T cell immunobiology. It was clonally expanded *in vivo*, resided within a T_effector_ population, and utilises a highly complex TCR-δ rearrangement.

LES γδ TCR engages the underside of the EPCR α1–α2 domain, a highly unusual binding mode relative to αβ TCRs, in a CDR3γ/δ-dependent manner, suggesting that cognate ligand interaction during infection might drive adaptive differentiation.

EPCR antigenicity likely relies on increased surface expression and/or aberrant glycosylation after HCMV infection and in some cancers, potentially qualifying it as a prototypic cognate adaptive γδ TCR ligand.

## Significance

Human γδ T cells are increasingly recognised to harbour adaptive-like subsets; however, how this immunobiology operates is poorly understood. Studies on the prototypic adaptive-like LES γδ T cell clonotype indicates that its clonal amplification, differentiation, and CDR3-mediated γδ TCR recognition of the host-encoded ligand are quantitatively and/or qualitatively altered under microbial and non-microbial stress. These features likely underpin *in vivo* adaptive-like immunobiology, and have major implications for understanding and therapeutically harnessing γδ T cell responses to infection and cancer.

## Glossary

### Abbreviations

Butyrophilin and butyrophilin-like molecules (BTN/BTNLs): immunoglobulin superfamily proteins (e.g., BTN3A, BTN2A1) that modulate T cell responses; select BTN/BTNL pairs are implicated in γδ T cell selection and/or activation, including BTN3A and BTN2A1, which can stimulate human Vγ9Vδ2 T cell activation.
CD1d: a nonclassical MHC class I-like lipid-presenting molecule that loads glycolipids and presents them to invariant NKT (iNKT) cells.
Chimeric antigen receptor T (CAR-T) cells: patient- or donor-derived T cells engineered to express a CAR consisting of an antibody-derived ectodomain that binds to cell-surface targets independently of MHC, fused to intracellular signalling elements enabling ligand-dependent T cell activation and targeted cytotoxicity against malignant or pathogenic cells.
Clonotype: a T or B cell clone defined by its unique antigen-receptor sequence – typically CDR3 nucleotide/amino-acid sequence and V(D)J usage – reflecting a shared clonal origin.
HMBPP: (E)-4-hydroxy-3-methyl-but-2-enyl pyrophosphate: a potent microbial phosphoantigen (P-Ag) from the non-mevalonate or MEP (2-C-methyl-D-erythritol 4-phosphate) isoprenoid pathway that is used by many bacteria, but is absent in mammals; it strongly activates human Vγ9Vδ2 T cells.
Invariant natural killer T (iNKT) cells: cells bearing a semi-invariant TCR that recognises CD1d-presented glycolipids, enabling swift cytokine release and immunoregulatory functions.
Mucosal-associated invariant T (MAIT) cells: innate-like αβ T cells recognising MR1-presented microbial riboflavin metabolites; they rapidly produce cytokines and contribute to barrier-tissue antimicrobial defence
Major histocompatibility complex (MHC) proteins: polymorphic antigen-presenting molecules (classes I and II) that display peptide fragments to αβ T cell receptors, shaping adaptive immunity and self/non-self-discrimination.
MHC restriction: the requirement that αβ T cells recognise antigenic peptides only when bound to self MHC molecules, a specificity imposed during thymic selection.
MR1: a predominantly monomorphic MHC class I-related molecule that presents small-molecule metabolites (notably riboflavin pathway derivatives) to MAIT cells.
Pathogen-associated molecular patterns (PAMPs): conserved microbial structures (e.g., lipopolysaccharide, double-stranded RNA, unmethylated CpG) typically recognised by pattern-recognition receptors to trigger innate immune activation.
Phosphoantigen (P-Ag): a small, non-peptidic prenyl-pyrophosphate metabolite – for example, isopentenyl pyrophosphate (IPP), HMBPP – that activates human Vγ9Vδ2 T cells via sensing by BTN3A1/BTN2A1 in target cells, linking microbial/isoprenoid metabolism to rapid innate-like T cell responses.
Private TCR: a TCR sequence found only in an individual and in a single clone, reflecting a unique recombination/selection event.
Public TCR: a recurrent TCR sequence (or near-identical motif) independently shared across individuals, often arising from biased/convergent recombination and/or strong antigenic selection.
T cell receptor (TCR): a heterodimeric receptor (αβ or γδ) generated by somatic V(D)J recombination, which recognises antigens and initiates T cell activation.

## Outstanding questions

How is tolerance to self-antigens established in the human γδ T cell compartment? Does this involve intrathymic deletional tolerance or activation threshold modulation in/outside the thymus?

If peripheral tolerance is critical in limiting reactivity of γδ T cells bearing TCRs that can recognise autoantigens, then which mechanisms are involved?

Do *in vivo*-selected adaptive γδ TCRs converge on common pathways of biological significance in particular infectious or tumour settings, and if so, what are these axes of immunosurveillance?

Do the expanded private clonotypes characteristic of T_effector_ populations recognise a limited range of public antigens, or are adaptive γδ TCR ligands also private?

How structurally diverse are γδ TCR ligands recognised *in vivo*? Will the dimensions of TCR–ligand interactions comply with existing models of TCR triggering such as kinetic segregation?

To what extent do adaptive γδ T cell responses in infection focus on direct recognition of pathogen-encoded antigens, as opposed to recognition of pathogen-dysregulated self-antigens?

How commonly will γδ TCR-mediated dual reactivity to infection and cancer be observed? Does this reflect recognition of identical ligands, and if so, are they similarly altered in each scenario?

In what manner are adaptive γδ TCR self-ligands dysregulated during infection or cancer to break tolerance? Are such changes quantitative or qualitative, and what processes do these represent: altered localisation, post-translational modification, or association?

What cellular niches will adaptive γδ T cell responses focus on?

How do the dynamics of adaptive-like γδ T cell responses compare with conventional MHC-restricted αβ T cell adaptive responses?
